# Premature termination of inpatient eating disorder treatment: Does timing matter?

**DOI:** 10.1186/s40337-023-00934-5

**Published:** 2023-11-27

**Authors:** Sarah Smith, Kalam Sutandar, Blake Woodside

**Affiliations:** 1 Hospital for Sick Children, 555 University Avenue, Toronto, ON M5G 1X8 Canada; 2 Department of Psychiatry, University of Toronto, 25 Sheppard Ave West, Suite 300, Toronto, ON M2N 6S6 Canada; 3 Department of Psychiatry, University of Toronto, 250 College Street, Toronto, ON M5T 1R8 Canada

**Keywords:** Anorexia nervosa, Premature termination of treatment, Treatment drop-out, Emotional dysregulation

## Abstract

**Background:**

Premature termination of treatment is a serious problem in the treatment of eating disorders. Prior research attempting to differentiate patients who are able to complete treatment from those who terminate early has yielded mixed results. One proposed explanation for this is a failure to examine the time course of treatment termination. This study was designed to explore associations between baseline patient characteristics and timing of treatment termination.

**Methods:**

Participants were 124 eating disorder patients admitted voluntarily to the inpatient program at Toronto General Hospital between 2009 and 2015. At admission, all patients completed measures of eating disorder symptoms, eating disorder cognitions, depressive symptoms and emotional dysregulation. Body weight was measured weekly. Data analyses were completed using one-way ANOVAs and Chi Square tests.

**Results:**

Results showed significant associations between timing of treatment termination and eating disorder diagnosis, severity of eating disorder cognitions and severity of depressive symptoms. Post-hoc analyses revealed that patients who left treatment early had more severe depressive symptoms, eating disorder cognitions related to eating and difficulties engaging in goal directed behaviors when emotionally dysregulated.

**Conclusions:**

Patients who terminated inpatient treatment early in their admissions differ from patients who terminated later and those who completed treatment. These differences have potential clinical implications for the clinical management of patients with severe eating disorders requiring inpatient admission.

*Trial registration* This paper is not associated with a clinical trial.

**Supplementary Information:**

The online version contains supplementary material available at 10.1186/s40337-023-00934-5.

## Abstract (plain language summary)

Patients being unable to complete inpatient treatment is serious problem in the treatment of eating disorders. Prior research attempting to identify differences between patients who can complete treatment and those who cannot has had mixed results. This study was designed to explore whether patients who leave treatment at different times differ from each other. To do this we compared eating disorder symptoms, eating disorder thoughts, depressive symptoms and emotional regulation symptoms of patients who left treatment early (0–4 weeks), later (after 4 weeks but before completion) and those who completed treatment. Results showed that patients who left treatment early reported the most severe eating disorder beliefs and depressive symptoms. They also had the most difficulties engaging in goal directed behaviours when experiencing intense emotions. They were not found to have differences in body weights or rates of eating disorder behaviors (i.e. self-induced vomiting). These results suggest that patients who leave treatment early are the most unwell and may benefit from learning emotional regulation skills prior to, or early in, treatment.

**Supplementary Information:**

The online version contains supplementary material available at 10.1186/s40337-023-00934-5.

## Background

Premature termination of treatment is a serious problem in eating disorder treatment. Rates of premature termination in specialized inpatient, or residential, eating disorder treatment programs range from 20 to 51% [1]. Premature termination of treatment has been defined as patient, or provider, initiated discharge prior to weight restoration in prior research [1]. Clinically, leaving inpatient treatment prior to weight restoration has been associated with poorer longer-term outcomes including persistent eating disorder symptomology and higher rates of re-hospitalization [2]. Prior studies have attempted to identify patient characteristics associated with premature termination of treatment by exploring potential differences in the characteristics of eating disorder patients who complete inpatient treatment and those who do not. However, much of this research has had mixed results. Specifically, there have been inconsistent findings on associations between premature termination of treatment and eating disorder diagnoses, eating disorder symptoms, depressive symptoms or demographic factors [3]. One proposed explanation for this variation in findings is a failure to examine the time course of premature termination of treatment as factors that contribute to early termination of treatment may differ from those that contribute to later termination of treatment [3, 4].

Few studies to date have examined the potential differences between eating disorder patients who leave treatment early compared to those who leave later but before treatment completion. Kahn and Pike compared patients discharged below 80% of their ideal body weight (labeled early dropouts) to those discharged between 81 and 90% of their ideal body weight (labeled late dropouts) and found that patients discharged at lower body weights had more prior eating disorder admissions. In this study, treatment completion was defined as maintaining a body weight at or above 90% of ideal body weight for at least two weeks [5]. Similarly, Vandereycken and Vansteenkiste found that patients who terminated intensive inpatient treatment in the first month of their admission had histories of more engagement in outpatient care [6]. A larger study by Huas et al. (n = 601) found that patients with anorexia nervosa who left inpatient treatment during the first week of admission had lower desired weights than those who left later, used significant more tobacco, alcohol, and recreational drugs, had more frequent histories of attempted suicide and scored higher on the Symptom Checklist 90 (SCL-90) psychoticism and hostility subscales. These findings were hypothesized to reflect impulsivity [4]. Of note, in Huas et al.’s study just over a quarter of premature terminations of treatment occurred in the first week of admission. Prior research at the site of this study identified distinct periods of highest probability of premature treatment termination early and late in treatment but did not explore patient characteristics in relation to timing of treatment termination [7]. Specifically, the highest probabilities of treatment termination were observed in week 1 and weeks 14–15 of treatment. This study was designed to further explore potential differences between eating disorder patients who terminate inpatient treatment at different time points.

In the light of the proposal that impulsivity may play a role in premature termination of treatment [4], we hypothesized that impulsivity may represent emotional dysregulation in this population. There is an increasing body of evidence examining emotional dysregulation in patients with eating disorders. Much of this research builds on Gratz and Roemer’s conceptualization of emotional regulation as a process of identifying emotions, accepting them, controlling behavior despite negative emotions and using different strategies in different situations to manage emotions and achieve goals [8]. Clinically, patients with anorexia nervosa have been found to have more difficulties with emotional regulation and distress tolerance than healthy controls [9–11]. Yet the role of emotion regulation in this disease remains controversial [12]. Recent research has found conflicting results on the effect of weight, weight restoration and inpatient treatment on emotional dysregulation with one study reporting improvements in all components of emotion regulation upon completion of inpatient treatment [13] while another found no relationship between weight restoration and emotional regulation [14]. Similarly, conflicting results have been found when exploring the potential significance of weight or body mass index (BMI) and emotional dysregulation at admission to inpatient treatment [15, 16]. Specifically, one study found a correlation between low BMI and emotion control [15] while another did not [16].

No research to date has examined a potential association between emotional regulation and premature termination of eating disorder treatment. Hence, this study was also designed to explore the relationship of emotional regulation to timing of inpatient treatment termination.

### Study aims and hypotheses

This study had two aims. The first was to examine associations between baseline patient characteristics and timing of treatment termination by comparing individuals who terminate inpatient eating disorder treatment early in their admission to those who terminate later and those who complete treatment on a series of previously studied factors including demographic characteristics, eating disorder behaviors, eating disorder psychopathology (thoughts and attitudes) and depressive symptoms. The second was to explore the potential association of emotional dysregulation to timing of treatment termination.

To this end, we defined three primary baseline symptom domains—(i) eating disorder behaviors and psychopathology, (ii) depressive symptoms and (iii) difficulties with emotional dysregulation.

Our clinical experience of treating eating disorder patients in an inpatient setting is that many who leave early are very emotionally dysregulated and cannot tolerate the containment or structure of an intensive inpatient program. Building on the proposal of Huas et al. [4] that patients who leave treatment early in their admission are more impulsive, we speculated that what may appear to be an impulsive choice to terminate treatment prematurely may represent emotional dysregulation as in this instance patients may struggle to identify intense emotions and respond to them in ways that allow them to remain in treatment [8]. Likewise, many of our patients who tolerate the beginning of their inpatient admissions (i.e. the first few weeks) and then leave treatment prior to completion report being unable to tolerate weight restoration. Therefore, we speculated that these patients may have more severe eating disorder beliefs, particularly with regards to shape and weight. This led us to hypothesize that: (i) Patients who leave treatment early will have more difficulties with emotional dysregulation at admission then those who leave later and those who complete treatment and (ii) Patients who leave treatment later, but prior to completion, will have more severe eating disorder beliefs at admission than those who complete treatment.

## Methods

### Participants

This study was done using existing data on 124 patients admitted to the inpatient eating disorder program at Toronto General Hospital between 2010 and 2015. All met diagnostic criteria for Anorexia Nervosa (AN) or AN-like Eating Disorder Not Otherwise Specified (ED-NOS) according to the Diagnostic and Statistical Manual of Mental Disorders, Fourth Edition, Text Revision (DSM-IV-TR). All admissions were voluntary and all patients consented to participate in ongoing research. There were 189 admissions in the study period for which psychometric inventories were available for 148 admissions (78.31%). For patients who had multiple admissions during the study period, only data from their first admissions was used limiting the sample to 124 patients. Patients who did not complete psychometric inventories at admission did not differ from those who did in terms of gender, age, diagnosis, body mass index (BMI) or number of prior inpatient admissions.

### Intervention

The inpatient eating disorders program at Toronto General Hospital operated as a multi-disciplinary intensive group therapy program focused on medical stabilization, weight restoration and the normalization of eating behaviors.

Care was provided by an interdisciplinary team including psychiatrists, psychologists, dieticians, social workers, nurses and occupational therapists. Patients participated in meal support, group and individual therapy and psychosocial rehabilitation. As patients stabilized medically, they were granted day and weekend passes and could eventually transitioned to day attendance. Patients were considered to have completed the inpatient program when they attained a healthy body weight operationalized as a Body Mass Index (BMI) of 20 kg/m. This definition of treatment completion was chosen to be consistent with prior studies on treatment outcome completed at the site of this study [7, 17] as well as international research [18].

Treatment could be terminated prematurely by the patient or treatment team. Patients could choose to leave the program at any time if they were medically stable. They could also be discharged by staff for not participating in the program (i.e., not participating in therapeutic tasks). Staff initiated premature terminations of treatment typically involved a long period of discussion about difficulties participating in the program and unsuccessful attempts to change the behaviors before a decision was made about discharge.

### Measures

Patients completed a standardized battery of psychological measures at admission including an Eating Disorder Examination (EDE), an Eating Disorder Examination Questionnaire (EDEQ), a Beck Depression Inventory (BDI) and a Difficulties in Emotion Regulation Scale (DERS).

Eating disorder behavioral frequencies were measured using the Eating Disorder Examination (EDE)—twelfth edition [19]. This a semi-structured diagnostic interview that assesses dietary restraint, frequency of eating disorder symptoms and degree of concern about shape and weight. It has been shown to have good validity, internal consistency [20] and inter-rater reliability [21]. Measures derived from this interview included the frequency of binge eating, self-induced vomiting (purging), laxative use, diuretic use and exercise. These items were selected from the EDE interview because prior research has shown that patient perceptions of binge eating differ from clinicians’ [22]. Patients also completed an Eating Disorder Examination Questionnaire (EDEQ) fourth edition [22]. This is a 31 item self-report questionnaire that assesses eating disorder symptoms on a series of Likert scales. It has four subscales: Shape Concern, Weight Concern, Eating Concern and Restraint. A global score can also be calculated. In this study, the total score was the metric selected as its’ internal consistency, test-test reliability [23] and construct validity have been established [22].

Depressive symptoms were measured using the Beck Depression Inventory II (BDI-II) [24]. The BDI is a 21-item self-report questionnaire. Responses to all items are added to create a total score. The BDI is one of the most commonly used research measures of depression and has been shown to have high internal consistency, construct validity [25] and adequate test–retest reliability [26]. It has also been used in prior research to measure depressive symptoms in eating disorder patients [7].

Emotional dysregulation was quantified using the Difficulties in Emotion Regulation Scale (DERS). The DERS is a 36 item self-report questionnaire that measures multiple aspects of emotional dysregulation. It has six subscales and a total score. The six subscales are i. Clarity (a lack of emotional clarity), ii. Awareness (a lack of emotional awareness), iii. Non-acceptance (non-acceptance of emotions), iv. Impulse (difficulties with impulse control), v. Goals (difficulties engaging in goal directed behavior when feeling negative emotions) and vi. Strategies (limited access to emotional regulation strategies) [8]. Prior examination of the psychometric qualities of the DERS has shown high internal consistency, good test–retest reliability and adequate construct validity [8]. The total DERS score was used as our baseline symptom domain in this study.

Height and weight were measured weekly throughout each patients’ admission and used to calculate Body Mass Index (BMI), weight gain and weekly rate of weight gain.

### Statistical analyses

All statistical analyses were conducted in SPSS Version 24 (SPSS Inc, Chicago). Patients were divided into three groups depending on the timing and outcome of treatment: treatment completers, early treatment terminations and late treatment terminations. Patients who remained in the program until achieving a BMI of 20 were considered completers; patients who left treatment in the first four weeks were classified as early treatment terminations and patients who left treatment after four weeks but before achieving a BMI of 20 were classified as late treatment terminations. These groups were defined based prior research in this population showing distinct periods of higher probabilities of inpatient treatment termination early and late in treatment at weeks one to four and then weeks fourteen to sixteen [4, 6, 7].

To determine whether the three timing of treatment termination groups differed in age, duration of illness, body mass index or average frequency of binging, purging, excessive exercise, diuretic use or laxative use in the three months prior to admission, a series of one-way ANOVAS were used. As in prior research on this patient population [13], univariate outliers (z =  ± 3.29) on episodic behavioral symptom variables (episodes of binging, purging, laxative and diuretic use and exercise) were addressed by identifying them using z scores and replacement with the next highest value in the distribution that was not an outlier. This was done to reduce the distributional skewness of these behavioral eating disorder symptoms as they were not normally distributed. Chi square tests were used to determine whether the three groups differed for categorical variables including eating disorder diagnoses, gender or ethnicity. No corrections were made for missing data.

To test our hypotheses, the three timing of treatment termination groups were compared on all three primary baseline symptom domains– (i) eating disorder psychopathology (ii) depressive symptoms, and (iii) emotional dysregulation using three independent ANOVAs. In these analyses, timing of treatment termination was the independent (group variable) and severity of eating disorder psychopathology, depressive symptoms and emotional regulation at admission were dependent variables. We used the DERS total score as our measures of emotional regulation (hypothesis 1) and the EDEQ total score as our measure of eating disorder cognitions (hypothesis 2). These analyses were subjected to a Bonferroni correction. For results that were statistically significant (*p* < .016) or that had a p value less than .05, Tukey’s post-hoc analyses were completed to specify between group differences. To increase the generalizability of findings to clinical practice, exploratory subscale analyses were also included in the post-hoc analyses to better characterize potential between group differences.

## Results

Diagnostically, 123 patients met criteria for anorexia nervosa (AN) and 1 met criteria for anorexia nervosa like eating disorder not otherwise specified (EDNOS). Among those with AN, 56 (45.5%) met criteria for the restricting subtype of the illness (AN-R) and 67 (54.5%) met criteria for the binge purge subtype of the illness (AN-BP).

Patients ranged in age from age 18 to 63 years with an average age of 30.81 (SD = 11.43) years. One hundred and nineteen (96%) identified as female, three (2.4%) identified as male, one (0.8%) identified as transgender and one (0.8%) preferred not to disclose their gender. Ninety-eight (79.0%) identified as Caucasian or with a specific European nationality (i.e. Dutch), 1 (0.8%) identified as Black, eight (6.5%) identified as Asian, 2 (1.6%) identified as Hispanic, 3 (2.4%) identified as Indian, 2 (1.6%) identified as Middle Eastern, 7 (5.6%) identified in non-specific ways (i.e. Canadian) and 8 (6.5%) patients did not report their ethnicity, At the time of admission, patients’ average Body Mass Index (BMI) was 15.21 (SD = 1.71) and they had been unwell for an average of 10.53 (SD = 9.59) years. Preceding the study period, 27 (21.8%) individuals had had prior inpatient admissions to our inpatient program.

The average length of stay for all first admissions in the study period was 13.88 (SD = 7.30) weeks. Thirteen patients left treatment in the first four weeks of treatment (10.5%), thirty-one left after four weeks but prior to weight restoration (25.0%) and eighty (64.5%) completed treatment.

Patients in the early treatment termination group had an average inpatient length of stay of 2.26 (SD = 0.88) weeks; patients in the later treatment termination group had an average length of stay of 10.28 (SD = 6.19) weeks and patients in the treatment completion group had an average length of treatment of 17.17 (SD = 5.48) weeks.

Comparison of timing of treatment termination groups showed no statistical differences in baseline demographic variables including age at admission, duration of illness, BMI at admission or average frequency of excessive exercise prior to admission. Sixty-five (54.6%) of patients reported engaging in excessive exercise in the three months prior to admission. Significant differences in of binging, purging, diuretic or laxative use in the three months prior to admission were also not found for those with the binge purge subtype of anorexia (n = 67) (Table 1).

**Table 1:** Demographics of patients by timing of treatment termination

Variable	*n*	Early termination	Late termination	Completion	Significance
Age	118	35.17 (SD = 14.31)	30.33 (SD = 9.41)	30.32 (SD = 11.67)	*p* = .383
BMI	124	15.44 (SD = 1.88)	14.84 (SD = 2.07)	15.31 (SD = 1.52)	*p* = .389
Duration of illness (years)	117	10.23 (SD = 7.62)	12.01 (SD = 9.81)	10.00 (SD = 9.84)	*p* = .626
Mean monthly # days of excessive exercise in 3 m before treatment	119	1.50 (SD = 2.74)	3.34 (SD = 4.00)	2.99 (SD = 3.02)	*p* = .250
Mean weekly # of binge episodes in 3 m before treatment	63	3.07 (SD = 6.12)	3.73 (SD = 9.00)	4.67 (SD = 8.77)	*p* = .846
Mean weekly # of purge episodes in 3 m before treatment (BP subtype)	64	17.22 (SD = 20.05)	10.38 (SD = 15.75)	10.97 (SD = 13.48)	*p* = .449
Mean monthly # laxative use episodes in 3 m before treatment (BP subtype)	64	4.27 (SD = 5.26)	3.12 (SD = 4.55)	1.59 (SD = 3.13)	*p* = .121
Mean monthly # diuretic use episodes in 3 m before treatment (BP subtype)	64	0.00 (SD = 0.00)	2.27 (SD = 7.01)	0.37 (SD = 1.44)	*p* = .184

**Fig. 1:**
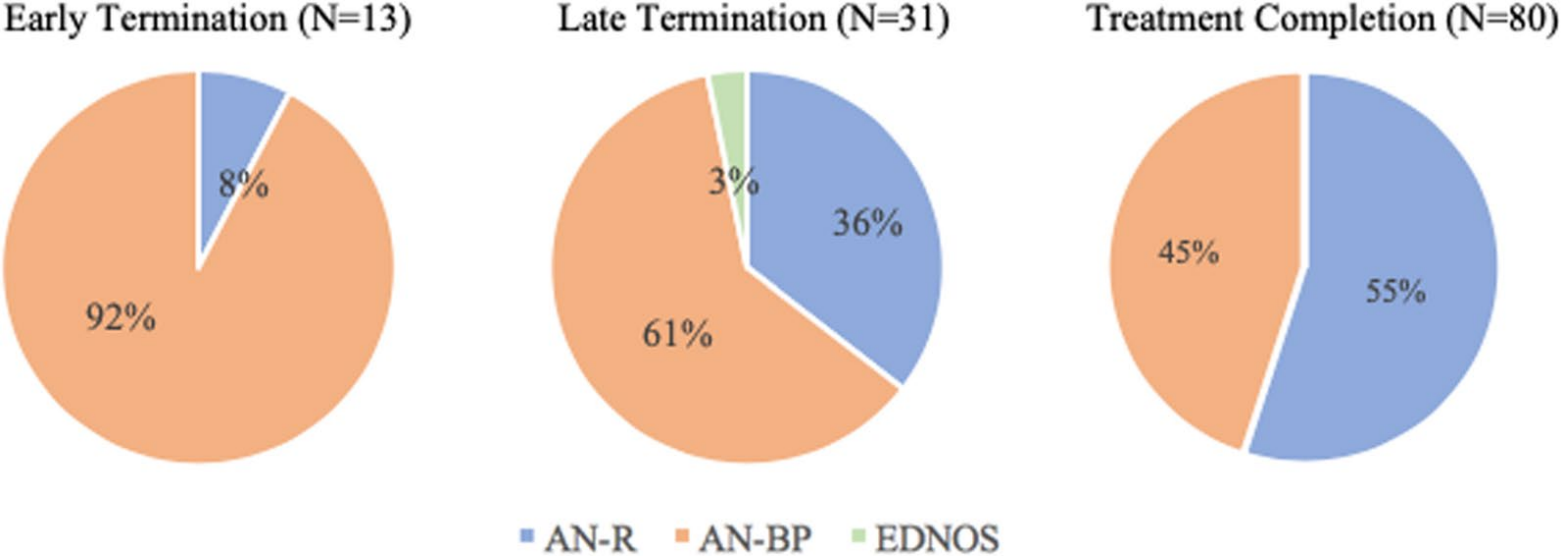
Timing of treatment termination and eating disorder diagnosis

There was a significant relationship between eating disorder subtype and timing of treatment termination (x^2^(4) = 14.43, *p* = .006) with patients leaving treatment early having the highest incidence of AN-BP diagnoses (92%) (Fig. 1).

There was no statistical difference in the frequency of identified genders, ethnicity or prior inpatient eating disorder admissions between the three treatment outcome groups.

Comparison of treatment outcome groups on our primary baseline symptom domains found significant differences. Timing of treatment termination was associated with severity of depressive symptoms at admission as measured by the BDI (F(2,120) = 5.83, *p* = .004) and severity of eating disorder psychopathology at admission as measured by the EDEQ total score (F(2,111) = 4.56, *p* = .013).

Difficulties with emotional regulation at admission as measured by the DERS total score did not achieve statistical significance (Table 2).

**Table 2:** Primary baseline symptom domains of patients by timing of treatment termination

Variable	*n*	Early termination	Late termination	Completion	Significance
BDI	123	47.62 (SD = 9.75)	37.00 (SD = 13.00)	34.44 (SD = 13.32)	*p* = .004**
EDEQ (total)	114	5.26 (SD = 0.60)	4.38 (SD = 1.16)	4.09 (SD = 1.42)	*p* = .013**
DERS (total)	115	143.83 (SD = 25.15)	117.233 (SD = 35.14)	119.12 (SD = 26.35)	*p* = .018*

*Significant at *p* < .05

**Significant at *p* < .016

Post hoc comparisons showed that patients who left treatment early had higher BDI scores (M = 47.61, SD = 9.75) that those who completed treatment (M = 34.44, SD = 12.32, *p* = .003). The BDI scores of those who left treatment earlier and later (M = 37.00, SD = 12.99, *p* = .038) did not differ statistically. The BDI scores of patients who left treatment later also did not differ significantly from those who completed treatment. BDI scores were available for all patients save one in the treatment completion group.

Patients who left treatment early had significantly higher EDEQ total scores (M = 5.26, SD = 0.60) than those completed their admissions (M = 4.09, SD = 1.42, *p* = .009) but not those who left later in treatment (M = 4.38, SD = 1.16). The EDEQ scores of patients who left treatment later also did not differ from those who completed treatment. EDEQ total scores were available for all 13 patients who terminated treatment early, 29 who terminated later but before completion and 72 who completed treatment.

Analyses of the overall DERS scores showed potential differences between patients who left treatment early (M = 143.83, SD = 25.15), those who left late (M = 117.233, SD = 35.14*, p* = .021) and those who completed treatment (M = 119.12, SD = 26.35, *p* = .019) that did not achieve statistical significance. Patients who left later did not differ from those who completed treatment. DERS total scores were available for all 13 patients who terminated treatment early, 30 who terminated later but before completion and 73 who completed treatment.

**Fig. 2:**
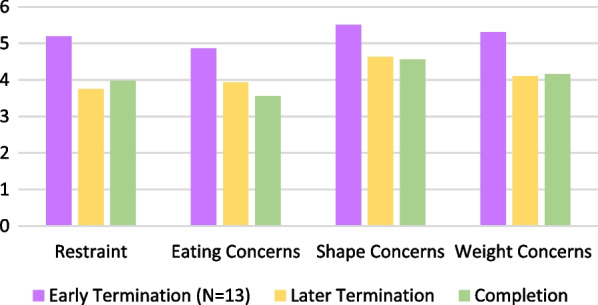
EDEQ subscale scores by timing of treatment termination

EDEQ subscale scores by timing of treatment termination are presented in Fig. 2. Results show that there was a statistically significant relationship between timing of termination of treatment and only one subscale: eating concerns. Patients who left treatment early had significantly higher scores on the eating concerns subscale (M = 4.86, SD = 0.92) than those who completed treatment M = 3.56, SD = 1.52, *p* = .014). Additional subscale comparisons are available in Additional file 1: Table S1.

**Fig. 3:**
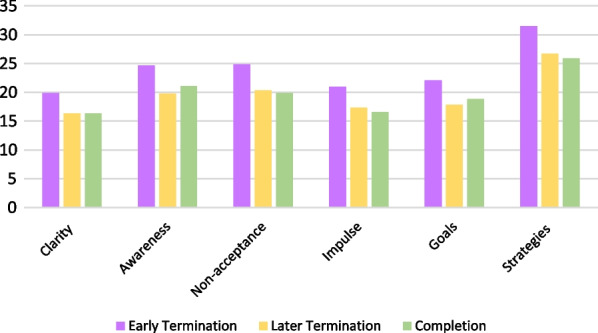
DERS Subscale scores by timing of treatment completion

DERS subscale scores are presented in Fig. 3. Patients who left treatment early had significantly higher scores on the goals subscale (M = 22.08, SD = 3.48) than those who left treatment later (M = 17.84, SD = 5.33, *p* = .014). Additional subscale comparisons were not statistically significant and are available in Additional file 1: Table S1.

## Discussion

The results of this study show patients who terminate inpatient eating disorder treatment early differ from those who terminate later and those who complete treatment.

Approximately a tenth (10.5%) of our sample left treatment in the first four weeks of their admission representing almost a third (29.5%) of treatment non-completers. This is a larger percentage than reported in the first month of treatment by one prior study [6] but comparable to the percentage of patients who terminated treatment in the first week of their admission in another prior, larger, study [4]. Differences in rates of premature termination between programs may represent differences in patient characteristics or provider practice patterns.

Patients who terminated treatment in the first four weeks of their admissions in our study had the highest total scores on all three of our primary outcomes—eating disorder psychopathology, depressive symptoms and emotional dysregulation. These patients’ total scores on the EDEQ and BDI were significantly higher compared to patients who completed treatment. Post-hoc analyses showed specific significant differences in the eating concerns subscale of the EDEQ. Total DERS scores did not achieve statistical significance with our adjusted alpha (< .016). However, differences in the DERS goals subscale were found when comparing early and late treatment terminations.

Patients who terminated treatment early were also more likely to have the binge purge subtype of anorexia but did not report more frequent eating disorder behaviors (binging, purging, laxative use, diuretic use or excessive exercise) or have lower body mass indexes than patients with the same diagnosis who terminated treatment later or were able to complete treatment. Nevertheless, their combination of more severe eating disorder psychopathology, more severe depressive symptoms and more severe emotional dysregulation suggests that these patients are the most unwell of those presenting for intensive, specialist eating disorder care, and that their symptoms them from other patients with the same eating disorder diagnoses accessing inpatient treatment.

Interestingly, our data suggests that the depressive (BDI) and eating disorder psychopathology (EDEQ) scores of patients with eating disorders severe enough to require inpatient admission appear to exist on a continuum (Table 2). Scores on our measure of emotional dysregulation do not follow this pattern, with the total scores of those who were able to complete treatment appearing similar to those who terminate treatment later in their admissions. This suggests that the most unique factor affecting early treatment termination may be higher levels of elements of emotional dysregulation. Although the overall DERS total score was not statistically significant, patients who terminated early did report significantly more difficulties engaging in goal directed behaviour when emotionally dysregulated. This is consistent with our hypothesis that emotional dysregulation affects whether patients can tolerate intensive inpatient care where they are required to engage in challenging situations including regular meals and therapy daily to achieve treatment goals including medical stabilization, weight restoration and the normalization of eating behaviors.

The differentiation of patients who terminate treatment early from those who terminate later and those who were able to complete treatment also provides a possible explanation for why prior research has reported inconsistent findings on the relationship between depressive symptoms. Several prior studies have found no relationship between treatment non-completion and depressive symptoms as measured by the self-report [4, 5, 27, 28], diagnostic interview [29] or existing comorbid diagnosis [30] while two studies have reported a significant effect of depressive symptoms measured by self-report [7] or diagnostic interview [31]. This study provides a possible explanation for these inconsistent findings as our post hoc analyses showed that while patients who terminate treatment early differ significantly from patients who complete treatment, patients who terminated treatment later but before completion did not differ significantly from those who were able to complete treatment. Thus, combining patients who terminated treatment early with those who terminated later in prior studies may have obscured important differences in depressive symptoms that only became evident when timing of premature termination of treatment was examined.

Similarly, the potential effects of specific eating disorder cognitions on treatment termination may have also been obscured by failing to examine the time course of premature treatment termination in prior research. Two prior studies reported no relationship between treatment completion and EDE [28] or EDEQ scores [32] while another study found a relationship between premature termination of treatment and higher scores on the EDE weight concern subscale as well as lower scores on the EDE restraint subscale [7]. The authors of the study that found a relationship between EDE scores and treatment completion hypothesized that their results could represent higher levels of impulsivity or independence as well as intolerance of weight restoration among patients who prematurely terminated treatment [7]. Our results partially support these possibilities, as patients who terminated treatment early did have higher reported levels of concern about eating at the time of admission than those who completed treatment but not significantly higher concerns about weight. Patients who terminated treatment later did not have significantly higher scores on any EDEQ subscales than those who completed treatment. Thus, our results do not support our second hypothesis that patients who tolerate early inpatient treatment and then leave treatment later but before completion have higher eating disorder psychopathology then patients who are able to complete treatment. One possible explanation for this is that patients who leave treatment later may develop more severe concerns about weight and shape only after beginning to gain weight—thus potential differences could not be evident in data collected at admission. Alternatively, patients with the most severe overall eating disorder psychopathology may be so entrenched in their illnesses that they could not tolerate even small changes in dietary intake resulting in early treatment termination before their body weight or shape are affected by treatment.

Diagnostically, our results are consistent with those of multiple prior studies that have shown a relationship between premature treatment termination and eating disorder diagnosis wherein patients with the binge-purge subtype of anorexia are less likely to complete treatment [5, 7, 27]. Our analyses support the finding of Woodside, Blackmore and Carter (2004) that patients with the binge purge subtype are not only likely to not complete treatment but the most likely to terminate inpatient treatment in the first four weeks after admission [7].

Importantly, this study is the first to examine the potential relationship between emotional dysregulation in premature termination of eating disorder treatment. Our results suggest that elements of emotional dysregulation may be related to timing of treatment termination clinically despite the DERS total score not achieving statistical significance. Specifically, our finding that patients who terminated treatment in the first few weeks of hospitalization reported the most difficulties engaging in goal directed behaviour when emotionally dysregulated suggests that a lack of emotional regulation skills affects individuals’ ability to tolerate intensive treatment. This finding is consistent with our clinical observation that patients who leave treatment early in their admissions do so because they are too emotionally dysregulated to tolerate our intensive inpatient program. It is also consistent with Gratz and Roemer’s model of emotional dysregulation [8]. It is possible that prior to admission these patients were using food restriction to regulate their emotions and without the possibility to continue using this maladaptive coping mechanism once admitted, became overwhelmed and unable to focus on their goals in treatment. This conceptualization is supported by research showing a relationship between lower body weight and lower ratings of emotional dysregulation in patients with anorexia nervosa [15]. It is also possible that elements of emotional dysregulation in these patients could manifest in impulsive decisions to leave treatment despite their treatment goals. Prior research on emotional dysregulation in intensive eating disorder treatment has shown decreases among treatment completers and no significant differences in emotional dysregulation comparing treatment completers and non-completers at admission. That research did not examine the timing of treatment termination [13]. Thus, our observation that patients who terminate treatment early exhibits a higher element of emotional dysregulation than those who those who leave later and those who complete is novel and suggests that being unable to focus on goals when dysregulated is an early barrier to engaging in treatment. In contrast to prior recommendations that intensive eating disorder treatment focus first weight restoration and then emotional regulation, our results raise the possibility there is a subset of patients, our early treatment terminators, who may require the opposite—treatment focusing on elements of emotional regulation skills and distress tolerance to allow them to tolerate the beginning of normalization of eating and weight restoration.

Clinically, potential applications of our findings include a pre-admission program focused on addressing depressive symptoms and elements of emotional dysregulation. This could include therapeutic content about distress tolerance, healthy coping skills and future goals or a more intensive focus on these elements in the first few days of patients’ inpatient admissions to attempt to decrease early premature termination of treatment. These skills may also help patients tolerate the distress of eating which was reflected in their high EDEQ scores or engage in treatment despite their more severe depressive symptoms. Given that our post hoc analysis revealed that patients who left treatment early differed from those who left later on the DERS subscale representing difficulties engaging in goal-directed behavior when upset, this domain of emotional regulation should be of particular foci in either application. As improvements in emotional regulation during treatment have also been associated with improvements in eating disorder psychopathology above and beyond the effect of weight restoration [13], an increasing focus on elements of emotional regulation or distress tolerance skills may also be beneficial for other patients requiring intensive inpatient eating disorders treatment in the future.

### Limitations

This study has several limitations. First, as the treating program had a limited number of inpatient eating disorder beds during the study period, our sample size is limited although comparable to many other eating disorder studies of premature termination of treatment [6, 7, 28, 31]. This small sample size did affect the statistical power of our analyses, especially of the relationships between timing of treatment termination and our three primary baseline symptom domains. It is possible that in a larger sample, emotional dysregulation may have attained statistical significance. Second, all analyses were done on existing data collected at admission to inpatient treatment. Details of the quality control of measures at the time of collection is not available. It is possible data collection at the time of termination of treatment may have been different from baseline measures at admission as nutritional rehabilitation has been associated with improvements in depressive symptoms [33, 34] and emotional regulation [13]. Future research on factors impacting premature termination of treatment should attempt to study larger samples of patients and collect data longitudinally to control for potential changes in symptoms in treatment and to better correlate symptoms to timing of treatment termination. Such research could also examine the relative importance of different patient characteristics as predictors of treatment outcome by employing statistical analyses, such as regression models, that would allow them to control for confounding.

## Conclusion

The findings of this study support the conclusion that patients who terminate inpatient eating disorder treatment early differ from those who terminate later and those who are able to achieve weight restoration. These individuals represent a distinct sub-group of patients characterized by more severe eating disorder psychopathology and depressive symptoms, as well as specific emotional regulation difficulties. Developing pre-admission, or early admission interventions, to better address their distress may be an important way to decrease rates of premature treatment termination and to improve treatment outcomes in specialized inpatient eating disorder care.

### Supplementary Information

**Additional file 1: Table S1.** EDEQ subscale scores by timing of termination of treatment. **Table S2.** DERS subscale scores by timing of termination of treatment.

## Data Availability

The datasets generated and/or analyzed during the current study are not publicly available due to containing patient data but may be available from the corresponding author on reasonable request.

## Abbreviations

AN: Anorexia nervosa
ED-NOS: Anorexia nervosa like eating disorder not otherwise specified
AN-R: Anorexia nervosa-restricting subtype
AN-BP: Anorexia nervosa-binge purge subtype
ANOVA: Analysis of variance
BDI: Beck depression inventory
BMI: Body mass index
DERS: Difficulties in Emotion Regulation Scale
DSM-IV-TR: Diagnostic and Statistical Manual of Mental Disorders, Fourth Edition, Text Revision
EDE: Eating Disorder Examination
EDEQ: Eating Disorder Examination Questionnaire
SCL-90: Symptom Checklist 90

## References

[1] Wallier J, Vibert S, Berthoz S, Huas C, Hubert T, Godart N (2009). Dropout from inpatient treatment for anorexia nervosa: critical review of the literature. Int J Eat Disord.

[2] Baran SA, Weltzin TE, Kaye WH (1995). Low discharge weight and outcome in anorexia nervosa. Am J Psychiatry.

[3] Fassino S, Piero A, Tomba E, Abbate-Daga G (2009). Factors associated with dropout from treatment for eating disorders: a comprehensive literature review. BMC Psychiatry.

[4] Huas C, Godart N, Foulon C (2011). Predictors of dropout from inpatient treatment for anorexia nervosa: data from a large French sample. Psychiatry Res.

[5] Kahn C, Pike KM (2001). In search of predictors of dropout from inpatient treatment for anorexia nervosa. Int J Eat Disord.

[6] Vandereycken W, Vansteenkiste M (2009). Let eating disorder patients decide: Providing choice may reduce early drop-out from inpatient treatment. Eur Eat Disord Rev.

[7] Woodside DB, Carter JC, Blackmore E (2004). Predictors of premature termination of inpatient treatment for anorexia nervosa. Am J Psychiatry.

[8] Gratz KL, Roemer L (2004). Multidimensional assessment of emotion regulation and dysregulation: development, factor structure, and initial validation of the difficulties in emotion regulation scale. J Psychopathol Behav Assess.

[9] Brockmeyer T, Skunde M, Wu M (2014). Difficulties in emotion regulation across the spectrum of eating disorders. Compr Psychiatry.

[10] Corstorphine E, Mountford V, Tomlinson S, Waller G, Meyer C (2007). Distress tolerance in the eating disorders. Eat Behav.

[11] Harrison A, Sullivan S, Tchanturia K, Treasure J (2009). Emotion recognition and regulation in anorexia nervosa. Clin Psychol Psychother.

[12] Haynos AF, Fruzzetti AE (2011). Anorexia nervosa as a disorder of emotion dysregulation: evidence and treatment implications. Clin Psychol Sci Pract.

[13] Rowsell M, MacDonald DE, Carter JC (2016). Emotion regulation difficulties in anorexia nervosa: associations with improvements in eating psychopathology. J Eat Disord.

[14] Haynos AF, Roberto CA, Martinez MA, Attia E, Fruzzetti AE (2014). Emotion regulation difficulties in anorexia nervosa before and after inpatient weight restoration. Int J Eat Disord.

[15] Brockmeyer T, Holtforth MG, Bents H, Kammerer A, Herzog W, Friederich HC (2012). Starvation and emotion regulation in anorexia nervosa. Compr Psychiatry.

[16] Racine SE, Wildes JE (2013). Emotion dysregulation and symptoms of anorexia nervosa: the unique roles of lack of emotional awareness and impulse control difficulties when upset. Int J Eat Disord.

[17] Carter JC, Bewell C, Blackmore E, Woodside DB (2006). The impact of childhood sexual abuse in anorexia nervosa. Child Abuse Negl.

[18] Pham-Scottez A, Huas C, Perez-Diaz F (2012). Why do people with eating disorders drop out from inpatient treatment? The role of personality factors. J Nerv Ment Dis.

[19] Fairburn CGCZ, Fairburn CGWG (1993). The eating disorder examination. Binge eating: nature, assessment and treatment.

[20] Cooper Z, Cooper PJ, Fairburn CG (1989). The validity of the eating disorder examination and its subscales. Br J Psychiatry J Ment Sci.

[21] Cooper Z, Fairburn C (1987). The Eating Disorder Examination: a semi-structured interview for the assessment of the specific psychopathology of eating disorders. Int J Eat Disord.

[22] Fairburn CG, Beglin SJ (1994). Assessment of eating disorders: Interview or self-report questionnaire?. Int J Eat Disord.

[23] Luce KH, Crowther JH (1999). The reliability of the Eating Disorder Examination-Self-Report Questionnaire Version (EDE-Q). Int J Eat Disord.

[24] Beck AT SR, Brown GK. Beck depression inventory-II (BDI-II). In: Corporation TP, editor. Toronto Harcourt Brace; 1996.

[25] Richter P, Werner J, Heerlein A, Kraus A, Sauer H (1998). On the validity of the Beck Depression Inventory. A review. Psychopathology.

[26] Wang YP, Gorenstein C (2013). Psychometric properties of the Beck Depression Inventory-II: a comprehensive review. Braz J Psychiatry.

[27] Surgenor LJ, Maguire S, Beumont PJV (2004). Drop-out from inpatient treatment for anorexia nervosa: Can risk factors be identified at point of admission?. Eur Eat Disord Rev.

[28] Dalle Grave R, Calugi S, Brambilla F, Marchesini G (2008). Personality dimensions and treatment drop-outs among eating disorder patients treated with cognitive behavior therapy. Psychiatry Res.

[29] Hartmann A, Wirth C, Zeeck A (2007). Prediction of failure of inpatient treatment of anorexia nervosa from early weight gain. Psychother Res.

[30] Masson PC, Perlman CM, Ross SA, Gates AL (2007). Premature termination of treatment in an inpatient eating disorder programme. Eur Eat Disord Rev.

[31] Zeeck A, Hartmann A, Buchholz C, Herzog T (2005). Drop outs from in-patient treatment of anorexia nervosa. Acta Psychiatr Scand.

[32] Sly R, Morgan JF, Mountford VA, Lacey JH (2013). Predicting premature termination of hospitalised treatment for anorexia nervosa: the roles of therapeutic alliance, motivation, and behaviour change. Eat Behav.

[33] Meehan KG, Loeb KL, Roberto CA, Attia E (2006). Mood change during weight restoration in patients with anorexia nervosa. Int J Eat Disord.

[34] Pollice C, Kaye WH, Greeno CG, Weltzin TE (1997). Relationship of depression, anxiety, and obsessionality to state of illness in anorexia nervosa. Int J Eat Disord.

