# Supplementary material for: Premature termination of inpatient eating disorder treatment: Does timing matter?
Source: J Eat Disord. 2023 Nov 27;11:210. doi: 10.1186/s40337-023-00934-5 (PMC10680217; doi:10.1186/s40337-023-00934-5)
Supplement: Supplementary file 1 — Additional file 1: Table S1. EDEQ subscale scores by timing of termination of treatment. Table S2. DERS subscale scores by timing of termination of treatment. [file 40337_2023_934_MOESM1_ESM.docx]

***Supplementary Materials***

*Supplementary Table 1: EDEQ Subscale Scores by Timing of Termination of Treatment* _____________________________________________________________________________________________

Subscale (n) Early Termination Late Termination Completion Significance

Restraint (123) 5.18 (SD = 0.89) 3.75 (SD = 1.96) -------------------- *p* = .033*

5.18 (SD = 0.89) --------------------- 3.98 (SD = 1.71) *p* = .054

--------------------- 3.75 (SD = 1.96) 3.98 (SD = 1.71) p = .791

Eating Concerns (120) 4.86 (SD = 0.92). 3.93 (SD = 1.70) --------------------- *p* = .159

4.86 (SD = 0.92). -------------------- 3.56 (SD = 1.52) *p* = .014**

-------------------- 3.93 (SD = 1.70) 3.56 (SD = 1.52) *p* = .495

Shape Concerns (119) 5.52 (SD = 0.67). 4.63 (SD = 1.39) - ------------------- *p* = .136

5.52 (SD = 0.67) --------------------- 4.56 (SD = 1.47) *p* = .061

--------------------- 4.63 (SD = 1.39) 4.56 (SD = 1.47) *p* = .973

Weight Concerns (124) 5.31 (SD = 0.82) 4.11 (SD = 1.77) -------------------- *p* = .066

5.31 (SD = 0.82) - ------------------- 5.31 (SD = 0.82) *p* = .047*

--------------------- 4.11 (SD = 1.77) 4.11 (SD = 1.77) *p* = .989

________________________________________________________________________________ _____________

**significant at p <.05, **significant <.016*

*Supplementary Table 2: DERS Subscale Scores by Timing of Termination of Treatment*

______________________________________________________________________________

Subscale (n) Early Termination Late Termination Completion Significance

Clarity (123) 19.85 (SD = 3.83) 16.35 (SD=5.48) --------------------- p = .071

19.85 (SD = 3.83) --------------------- 16.33 (SD=4.58) p = .039 * ---------------------- 16.35 (SD=5.48) 16.33 (SD=4.58) p = 1.00

Awareness (117) 24.67 (SD = 4.64) 19.73 (SD = 5.97) --------------------- p = .022*

24.67 (SD = 4.64) --------------------- 21.12 (SD = 5.24) p = .090

--------------------- 19.73 (SD = 5.97) 21.12 (SD = 5.24). p = .460

Non-acceptance (117) 24.83 (SD = 6.39) 20.35 (SD = 8.02) ---------------------- p = .173

24.83 (SD = 6.39) ---------------------- 19.89 (SD = 7.12) p = .080

---------------------- 20.35 (SD = 8.02) 19.89 (SD = 7.12). p = .953

Impulse (119) 21.00 (SD = 5.89) 17.35 (SD = 7.07) --------------------- p = .250

21.00 (SD = 5.89) --------------------- 16.58 (SD = 6.66) p = .089

--------------------- 17.35 (SD = 7.07) 16.58 (SD = 6.66) p = .850

Goals (119) 22.08 (SD = 3.48) 17.84 (SD = 5.33) --------------------- p = .014*

22.08 (SD = 3.48) --------------------- 18.82 (SD = 4.08) p = .047

--------------------- 17.84 (SD = 5.33) 18.82 (SD = 4.08) p = .551

Strategies (119) 31.50 (SD = 7.28) 26.71 (SD = 9.36) --------------------- p = .208

31.50 (SD = 7.28) --------------------- 25.89 (SD = 7.92) p = .078*

--------------------- 26.71 (SD = 9.36) 25.89 (SD = 7.92) p = .889

_________________________________________________________________________________________

**significant at p <.05, **significant <.016*
